# Self-Conscious Affect Is Modulated by Rapid Eye Movement Sleep but Not by Targeted Memory Reactivation–A Pilot Study

**DOI:** 10.3389/fpsyg.2021.730924

**Published:** 2021-12-13

**Authors:** Risto Halonen, Liisa Kuula, Tommi Makkonen, Jaakko Kauramäki, Anu-Katriina Pesonen

**Affiliations:** ^1^Sleepwell Research Program, Faculty of Medicine, University of Helsinki, Helsinki, Finland; ^2^Cognitive Brain Research Unit, Faculty of Medicine, University of Helsinki, Helsinki, Finland

**Keywords:** targeted memory reactivation, REM sleep, REM fragmentation, shame, skin conductance response, embarrassment, affective habituation, slow-wave sleep

## Abstract

The neurophysiological properties of rapid eye movement sleep (REMS) are believed to tune down stressor-related emotional responses. While prior experimental findings are controversial, evidence suggests that affective habituation is hindered if REMS is fragmented. To elucidate the topic, we evoked self-conscious negative affect in the participants (*N* = 32) by exposing them to their own out-of-tune singing in the evening. Affective response to the stressor was measured with skin conductance response and subjectively reported embarrassment. To address possible inter-individual variance toward the stressor, we measured the shame-proneness of participants with an established questionnaire. The stressor was paired with a sound cue to pilot a targeted memory reactivation (TMR) protocol during the subsequent night's sleep. The sample was divided into three conditions: control (no TMR), TMR during slow-wave sleep, and TMR during REMS. We found that pre- to post-sleep change in affective response was not influenced by TMR. However, REMS percentage was associated negatively with overnight skin conductance response habituation, especially in those individuals whose REMS was fragmented. Moreover, shame-proneness interacted with REM fragmentation such that the higher the shame-proneness, the more the affective habituation was dependent on non-fragmented REMS. In summary, the potential of REMS in affective processing may depend on the quality of REMS as well as on individual vulnerability toward the stressor type.

## Introduction

Understanding how sleep can promote offline emotional processing opens up new perspectives for the concept of emotion regulation. For example, evidence points to poorer downregulation of emotional distress overnight in individuals with insomnia (Wassing et al., 2019b). The neurochemical conditions and activity patterns specifically during rapid eye movement sleep (REMS) are suggested to depotentiate the affective strength of memories *via* repeated limbic circuit activations (Walker and Van Der Helm, 2009; Goldstein and Walker, 2014). However, experimental support is equivocal. While some evidence indicates that REMS attenuates the reactivity toward emotional stimuli (Gujar et al., 2011; Rosales-Lagarde et al., 2012; Wassing et al., 2019a), this view is challenged by numerous reports showing REMS to be associated with elevated post-sleep electrodermal response (Pace-Schott et al., 2011; Gilson et al., 2015; Werner et al., 2015) and higher subjectively evaluated affect (Lara-Carrasco et al., 2009; Gilson et al., 2015; Werner et al., 2020).

The missing piece in this puzzle may be the quality of REMS. Disruptions of REMS can precede the onset of pathological conditions such as post-traumatic stress disorder (PTSD), where the emotional memories fail to dissipate during sleep (Pace-Schott et al., 2015). Additionally, the fragmentation of REMS is shown to disrupt the overnight processing of emotional distress (Wassing et al., 2016, 2019a), and it is also associated with more depressive symptoms and with a genetic propensity for depression (Pesonen et al., 2019). Conversely, a stronger overnight decrease in amygdala reactivity is observed with an increased duration of unperturbed REMS (Wassing et al., 2019a). However, disrupted slow-wave sleep (SWS) is also shown to impair mood (Finan et al., 2015), suggesting that emotional regulation is not limited to REMS.

An approach to directly modulate the content of sleep-driven processing is called targeted memory reactivation (TMR). Typically, in TMR, the processed material (unconditioned stimulus, UCS) is paired with a sensory stimulus such as an odor or a sound (conditioned stimulus, CS). During subsequent sleep, the person is exposed to the CS to reactivate the associated memory representation (Hu et al., 2020). While the potential of TMR during SWS on declarative memory improvement is acknowledged (Hu et al., 2020), attempts to enhance affective habituation with REMS-linked TMR are emerging. In a recent study, sound-cued TMR during REMS promoted the habituation of subjective arousal responses toward negative images (Hutchison et al., 2021). Another study focused on negative self-conscious emotion, having the participants listen to a recording of their own out-of-tune singing. The stressor was cued with an odor, and exposure to the odor during REMS attenuated the post-sleep amygdala reactivity of the participants toward the stressor (Wassing et al., 2019a).

In this study, we exposed the participants to self-conscious affect by having them listen to a playback of their own singing, a stimulus shown to cause shame and embarrassment (Wassing et al., 2019a). Piloting TMR, we paired the playback with a sound cue and examined how both physiological and subjective embarrassment were affected by TMR applied during REM or SWS. Additionally, we investigated how the proportion and fragmentation of both REMS and SWS are related to affective habituation. Finally, recognizing that the propensity for self-conscious affect may modulate autonomic responsivity (Hofmann et al., 2006), we also explored whether trait-like shame-proneness is associated with affective reactivity and its overnight habituation.

## Methods

### Participants

Participants were recruited by word of mouth among the contacts of the research group and voluntary students from psychology courses. During the study, the participants were only in contact with a non-familiar experimenter. Exclusion criteria were any self-reported diagnosed sleep, mood, or anxiety disorder, the use of any medication that could affect sleep, acute sickness, and gold allergy (polysomnography electrodes were gold-plated). One participant was excluded due to generalized anxiety disorder, and another due to prevalent sleep disturbances caused by hypothyroidism medication. The analytical sample size was *N* = 32, randomized into SWS (*n* = 11), REM (*n* = 10), and control groups (*n* = 11). All participants were informed of the nature of the experiment in advance, and all gave written informed consent. The study was approved by the Ethics Committee of Helsinki University Central Hospital. All procedures followed were in accordance with the Declaration of Helsinki and its later amendments.

### Study Flow and TMR Protocol

**Figure 1:**
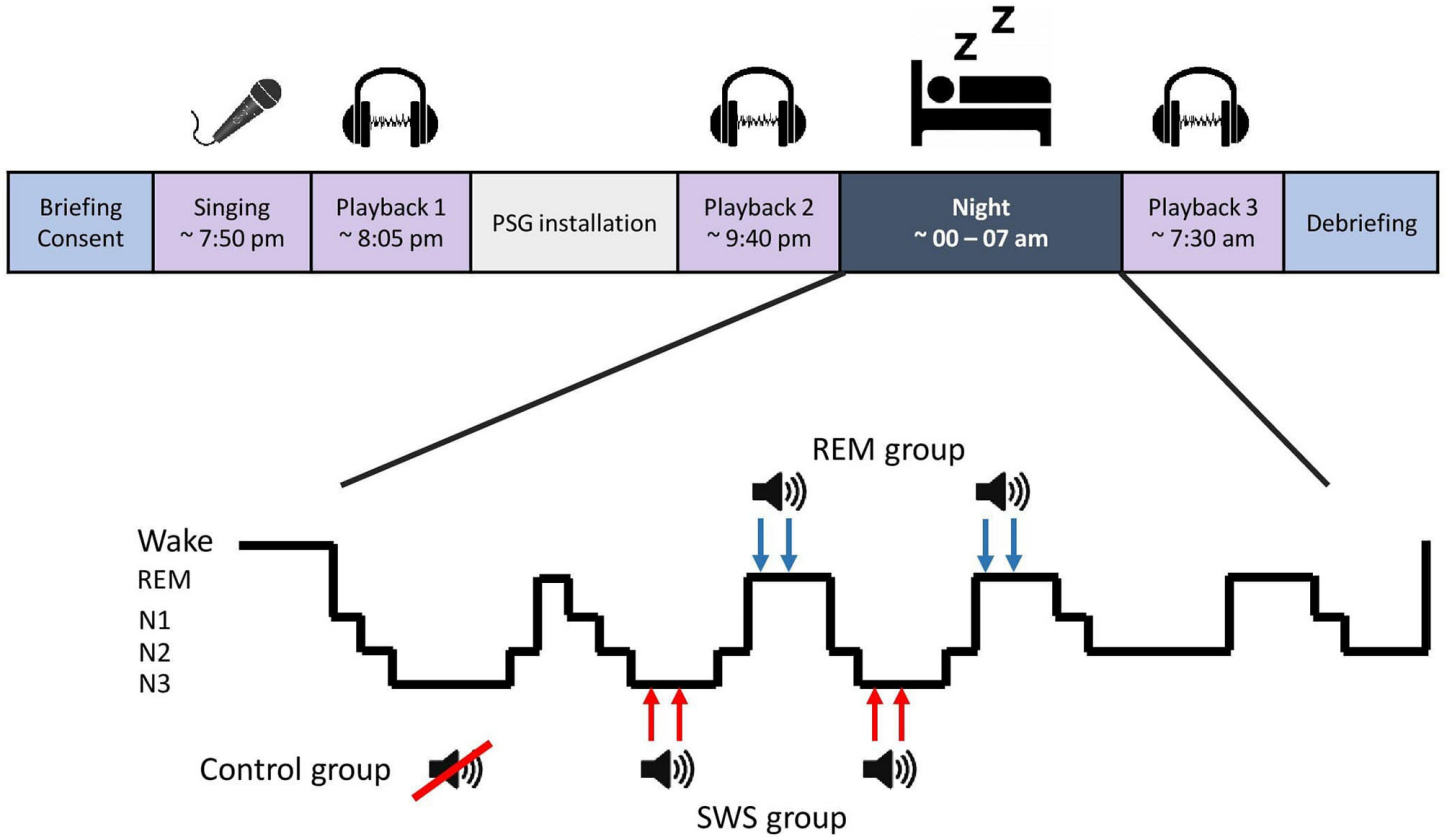
Study flow and targeted memory reactivation process.

The participants arrived at the laboratory in the early evening. After the briefing, they sang a karaoke version of Abba's Dancing Queen without hearing their own voice through headphones, promoting out-of-tune singing. The singing was recorded, and the experimenter created a ~1-min compilation of the recording without background music (UCS) using Audacity 2.3.2 software. The compilation consisted of three selected clips with 5-s silent intervals between clips. A 300 Hz sine wave sound, 250 ms (CS), was inserted in both silent intervals and at the beginning and the end of the compilation. Soon after this, the participants listened to the compilation (Playback 1), and the affective response was measured (refer to section Affective Response). The experimenter ensured that the participants had noticed the CS within the compilation. Polysomnography (PSG) was then attached, and before bedtime, Playback 2 took place. During sleep, TMR was applied for SWS and REM groups during their respective sleep stages. Aiming at the second and third sleep episode, the CS was played 12 times with 1.5-s intervals *via* a speaker in the bedroom (40 dB; reduced by 5 dB if arousal emerged), two times per sleep episode (i.e., four rounds, 78 s of cueing in total). After awakening and morning routines, Playback 3 was conducted. Refer to Figure 1 for a process overview.

### Affective Response

We assessed objective and subjective affective responses at Playbacks 1–3 (Figure 1). Objective response, i.e., skin conductance level (SCL), was measured from middle and ring fingers of the non-dominant hand using a galvanic skin sensor, connected to a Brain Products QuickAmp amplifier (Brain Products GmbH, Gilching, Germany). SCL was recorded at a 500 Hz sampling rate and analyzed using Matlab R2018a.

Baseline SCL was recorded from the participant sitting in a quiet room for 5 min and averaging over the period. Next, the participants listened to the UCS *via* headphones in the presence of the experimenter, and the SCL during the playback was measured and averaged. Skin conductance response (SCR) was the percentual difference between the baseline and playback SCLs. To represent an average electrodermal response of a person over the three playback occasions we calculated a mean over SCR_1_, SCR_2_, and SCR_3_ (SCR_Mean_).

Subjective embarrassment (Emb) was verbally asked after each playback with questions: (1) “How ashamed did you feel during the playback?” and (2) “How stressful was it to listen to the playback?” on a scale from 0 to 4 (i.e., none to highly). The mean of these values denoted the Emb value. The mean of Emb_1_, Emb_2_, and Emb_3_ (Emb_Mean_) represented the average self-reported embarrassment of a person. Overnight affective habituation, i.e., response decrease, was calculated for objective and subjective responses as follows: SCR_3−_ SCR_2_ = SCR_Decr_; and Emb_3−_ Emb_2_ = Emb_Decr_.

### Polysomnography and Sleep Fragmentation

All recordings were performed using either SOMNOscreen plus or SOMNOscreen HD (SOMNOmedics GmbH, Germany). Gold cup electrodes were attached at six electroencephalography (EEG) locations (frontal hemispheres: F3, F4; central: C3, C4; occipital: O1, O2), and mastoids (A1, A2). The electrooculogram (EOG) and the electromyogram (EMG) were measured using disposable adhesive electrodes (Ambu Neuroline 715, Ambu A/S, Denmark), two locations for EOG and three for EMG. An online reference Cz and a ground electrode in the forehead were used. The sampling rate was 256 Hz (the hardware filters for SOMNOscreen plus are 0.2–35 Hz). PSG data were scored manually using the DOMINO program (v2.7; SOMNOmedics GmbH, Germany) in 30-s epochs into N1, N2, N3, REM, and wake, according to AASM guidelines (AASM Manual for the Scoring of Sleep and Associated Events). Arousals were also marked. The proportions of each sleep stage were calculated by dividing the time spent in a certain stage by total sleep time (i.e., N1, N2, N3, and REM%).

Rapid eye movement fragmentation (REM_Frag_) was defined as the time spent in either wake, N1, N2, or arousals during REMS episodes divided by REMS duration during the whole night. The first REM epoch denoted the start of a REM episode, and the episode was concluded with the start of at least 4 consecutive min of wake or non-REM. SWS episodes and fragmentation (SWS_Frag_) were defined otherwise similarly, but SWS episodes ended at the start of a REM episode, or 4 min of consecutive wake, N1 or N2.

### Questionnaires

To evaluate trait-like shame-proneness, the participants filled the Test of Self-Conscious Affect (TOSCA-3; Tangney et al., 2000). Shame-proneness comprised the sum of questions 1A, 2B, 3E, 4A, 5C, 6C, 7A, 8A, 9B, 10D, 11B, 12B, 13B, 14A, 15A, and 16C, i.e., TOSCA-3_Shame_. We also assessed self-reported sleep characteristics (Pittsburgh Sleep Quality Index, PSQI; Buysse et al., 1989), depression symptoms (Beck Depression Inventory, BDI; Beck et al., 1996), and generalized anxiety symptoms (Generalized Anxiety Disorder 7, GAD-7; Williams, 2014).

The participants were screened for their experience in performing and singing by using two questions, scaling from 1 to 5 (none to very much): “Previous performing experience, e.g., speeches, presentations, acting, or singing?” and “Do you sing at leisure time or work?” The mean score was denoted as “singing experience.”

### Statistical Analyses

We used one-way ANOVA to test whether the TMR subgroups differed in age, sleep measures, questionnaire scores, singing experience, or affective habituation. The sex ratio between the subgroups was tested with chi-squared. To test for differences in subjective response between the playback occasions, we used repeated measures ANOVA. The difference between baseline and playback SCLs across Playbacks 1–3 was tested with mixed ANOVA, i.e., 3X2 design (three occasions; baseline and playback). We used linear regression to examine the associations between mean affective responses and the questionnaire scores and between overnight affective habituation and REM%, REM_Frag_, SWS%, and SWS_Frag._ Two-way ANOVA was used to investigate how affective habituation was associated with the continuous-by-continuous interactions between REM% and REM_Frag_ or between SWS% and SWS_Frag_ as well as between TOSCA-3_Shame_ and REM%, REM_Frag_ SWS% or SWS_Frag_.

In all analyses on overnight affective habituation, we ran a raw model without covariates, and a control model controlling for sex, age, singing experience, and time spent awake between Playbacks 2 and 3. Statistically significant raw model results were re-tested with the control model.

The nominal level of statistical significance was set at *p* < 0.05. In TMR piloting we focused on effect sizes, expecting large η^2^ (>0.14), based on estimates from previous findings (Wassing et al., 2019a; Hutchison et al., 2021). Statistical analyses were performed using IBM SPSS Statistics for Windows, version 27.0 (IBM Corp., Armonk, NY, USA).

## Results

### Sample Characteristics

Table 1 presents the age, sleep measures, questionnaire scores, singing experience, and mean affective responses of the analytical sample (*N* = 32, 24 women). No significant differences were found between the N3, REM, and control subgroups (*p*-values ≥ 0.161). Sex ratio also did not differ between the subgroups (p_χ2_ = 0.471).

**Table 1:** Sample characteristics.

	**SWS group** **(***n*** = 11)**	**REM group** **(***n*** = 10)**	**Control group** **(***n*** = 11)**	
	**Mean (SD)**	**Mean (SD)**	**Mean (SD)**	***P***
Age (years)	28.5 (11.0)	28.5 (12.5)	25.2 (7.3)	0.70
Sleep duration (h:mm)	6:32 (1:14)	6:23 (0:58)	7:06 (0:45)	0.27
N1 (%)	7.5 (3.3)	11.1 (6.7)	8.5 (12.7)	0.62
N2 (%)	48.0 (5.6)	49.4 (6.2)	46.7 (9.7)	0.73
N3 (%)	23.2 (6.7)	21.9 (6.9)	23.3 (4.0)	0.85
REM (%)	21.3 (6.0)	17.7 (7.0)	21.5 (5.8)	0.31
REM fragmentation (%)	8.5 (6.6)	8.1 (5.0)	7.5 (6.2)	0.93
N3 fragmentation (%)	14.3 (9.0)	15.9 (10.1)	9.7 (7.2)	0.28
WASO (h:mm)	0:29 (0:39)	0:27 (0:28)	0:30 (0:29)	0.98
GAD-7	2.0 (1.8)	3.1 (2.7)	2.5 (2.2)	0.53
BDI	3.1 (3.1)	5.5 (5.2)	4.6 (4.5)	0.44
PSQI	7.6 (2.3)	7.3 (1.8)	7.4 (2.6)	0.94
TOSCA-3_Shame_	42.3 (10.6)	49.1 (7.8)	41.5 (10.2)	0.16
Singing experience	2.4 (0.6)	2.9 (0.7)	2.5 (0.5)	0.20
SCR_Mean_ (%)	28.6 (11.0)	35.6 (13.1)	27.6 (19.3)	0.46
Emb_Mean_	1.3 (1.0)	1.6 (0.8)	1.6 (1.1)	0.76

*SWS, slow wave sleep; REM, rapid eye movement; N1–N3%, The percentage of sleep stages non-REM 1–3; REM%, the percentage of REM sleep; WASO, wake after sleep onset; GAD-7, sum score of Generalized Anxiety Disorder 7 questionnaire; BDI, sum score of Beck Depression Inventory questionnaire; PSQI, Pittsburgh Sleep Quality Index score; TOSCA-3_Shame_, sum score of TOSCA-3 questionnaire's shame subscale; SCR_Mean_, average electrodermal response over the three playback occasions (n = 30 due to excluded outliers); Emb_Mean_, average self-reported embarrassment; SD, standard deviation; p, p-value of the difference between N3, REM, and control subgroups*.

### Preliminary Analyses

Due to technical issues, we lost the PSG data of one participant and morning SCR data of another, thus excluding these observations from the analyses on REMS parameters and overnight habituation. One participant's SCR_Mean_ exceeded the sample mean by 4.7 SDs, and another showed +3.8 SDs in SCR_Decr_. We excluded these participants from the SCR analyses. Thus, analytical samples concerning SCR_Mean_ and Emb_Mean_ numbered 30 and 32, respectively. Tests regarding sleep parameters and SCR_Mean_/Emb_Mean_ included 29/31 participants. TMR groups for SCR_Decr_/Emb_Decr_ were as follows: N3 10/11; REM 8/10; and control 11/11. Non-normally distributed REM_Frag_ (Kolmogorov–Smirnov; *p* = 0.006) prompted us to deploy square root transformation, significant findings examined also with the transformed variable (sqrt-REM_Frag_).

### Emotional Response and Questionnaires

**Figure 2:**
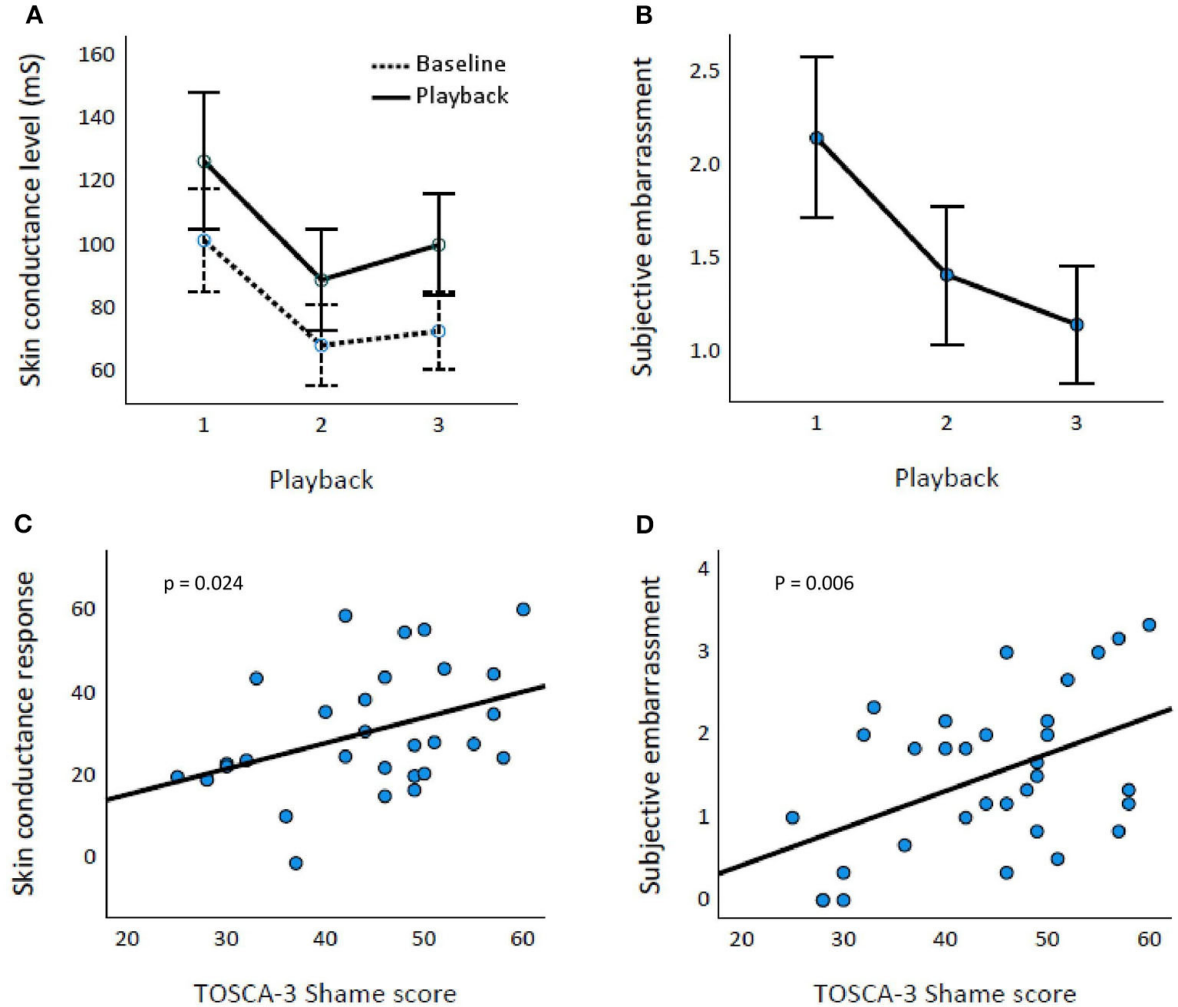
Affective response and TOSCA-3_Shame_ scores. Baseline and playback skin conductance levels **(A)** and subjective embarrassment **(B)** across Playbacks 1–3. Error bars represent 95% CIs. TOSCA-3_Shame_ score is associated with both skin conductance response (percentual increase; *p* = 0.024) **(C)** and subjective embarrassment (*p* = 0.006) **(D)**.

The time of the playback affected both SCR [Huynh–Feldt epsilon = 0.710, *F*_(1.42,54)_ = 12.099, *p* < 0.001; Figure 2A] and Emb [*F*_(2,62)_ = 32.813, *p* < 0.001] values (Figure 2B). The playback SCL differed significantly from the baseline SCL [*F*_(1,27)_ = 65.734, *p* < 0.001].

TOSCA-3_Shame_ was associated significantly with SCR_Mean_ [*t*_(1,28)_ = 2.385, *p* = 0.024] and Emb_Mean_ [*t*_(1,30)_ = 2.950, *p* = 0.006; Figures 2C,D, respectively]. BDI, GAD-7, or PSQI scores were not associated with SCR_Mean_ or Emb_Mean_ (*p*-values ≥ 0.075).

### Targeted Memory Reactivation

Of the cues, 90% hit the intended sleep stage. The deviations concerned the SWS group, where some cues given at a stage later were scored as N2. While all participants were not cued four times due to short sleep, the mean amount of cueing did not differ between the SWS and REM groups (64 and 72 s, respectively; *p* = 0.390). TMR condition (SWS, REM, or control) was not associated with differences in SCR_Decr_ [*F*_(2,26)_ = 0.012, *p* = 0.991, η^2^ = 0.001] or Emb_Decr_ [*F*_(2,28)_ = 0.149, *p* = 0.862, η^2^ = 0.010].

### REM, SWS, and Affective Habituation

**Figure 3:**
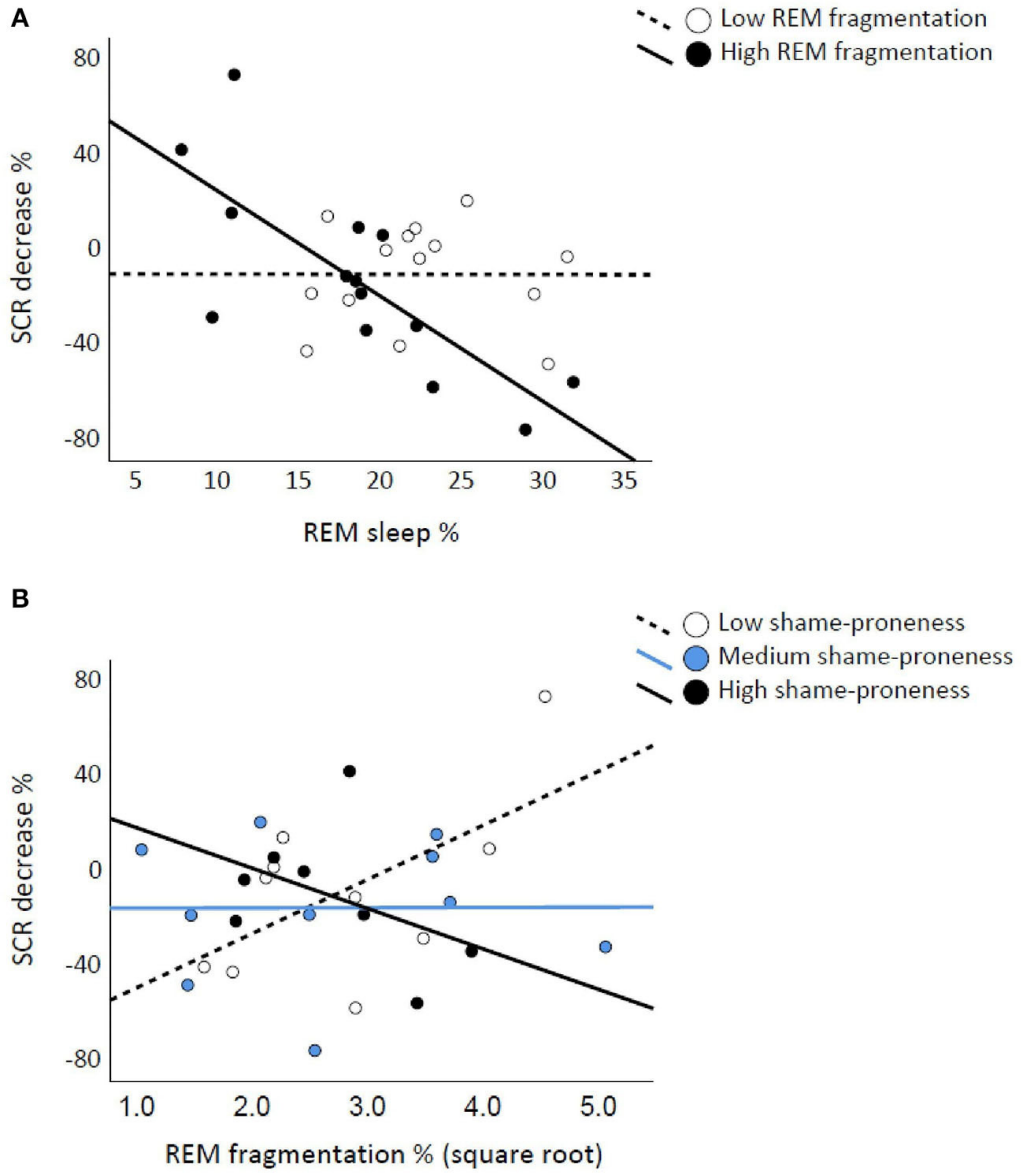
Rapid eye movement (REM) fragmentation interacts with REM percentage and TOSCA-3_Shame_ on skin conductance response (SCR) decreases. REM percentage is associated with lower SCR decrease if REM sleep is fragmented, the continuous-by-continuous interaction illustrated with median split **(A)**. Low and High shame-prone individuals show opposite associations between REM fragmentation (square root transformed) and SCR decrease, illustrated with tertile division **(B)**.

The SCR_Decr_ was associated negatively with REM% [*t*_(1,26)_ = −2.959, *p* = 0.00] but not with REM_Frag_ [*t*_(1,26)_ = 0.883, *p* = 0.386]. REM% and REM_Frag_ interacted significantly on SCR_Decr_ [*F*_(6,21)_ = 5.754, *p* = 0.025; control model *p* = 0.023; sqrt-REM_Frag_ *p* = 0.037]. The scatterplots in Figure 3A show that higher REM% is associated with less habituated SCR if REMS is fragmented. Emb_Decr_ was not associated significantly with REM% or REM_Frag_ or with their interaction (*p*-values ≥ 0.372). SWS%, SWS_Frag_, or their interaction was not significantly associated with SCR_Decr_ or Emb_Decr_ (*p*-values ≥ 0.300).

### Shame-Proneness, Sleep, and Affective Reactivity

With SCR_Decr_ as the dependent variable, the TOSCA-3_Shame_ score did not interact significantly with REM% [*F*_(3,24)_ = 0.195, *p* = 0.663]. However, “TOSCA-3_Shame_ X REM_Frag_” was significant [*F*_(4,24)_ = 5.083, *p* = 0.034; control model *p* = 0.041; sqrt-REM_Frag_ *p* = 0.047]. Figure 3B illustrates that fragmented REMS associates with attenuated SCR when shame-proneness is low, the pattern being opposite for highly shame-prone individuals. Regarding Emb_Decr_, TOSCA-3_Shame_ did not interact significantly with REM% or REM_Frag_ (*p*-values ≥ 0.292). Neither SWS% nor SWS_Frag_ interacted significantly with TOSCA-3_Shame_ on SCR_Decr_ or Emb_Decr_ (*p*-values > 0.436).

## Discussion

In this study, we piloted whether TMR during REMS or SWS influenced pre- to post-sleep habituation of SCR or subjective embarrassment, but no effect was found. We found that a higher proportion of REMS was associated with reduced habituation to stress. However, this association was specifically observed when REMS was fragmented. Additionally, we found that shame-proneness moderated the association between fragmented REMS and overnight habituation.

According to theoretical and experimental work (Walker and Van Der Helm, 2009; Gujar et al., 2011; Rosales-Lagarde et al., 2012; Wassing et al., 2019a), REMS depotentiates the affective load of memories. However, several contrary results indicate that REMS conserves pre-sleep reactivity over a sleep period (Pace-Schott et al., 2011; Gilson et al., 2015; Werner et al., 2015, 2020), which is supported by the present study. We saw a negative association between REMS percentage and the overnight change of playback-induced SCR. Investigating the quality of REMS is necessary. Fragmentation reportedly modulates the influence of REMS on emotional processing (Wassing et al., 2019a). In our study, REM fragmentation as such did not associate with emotional habituation, but it interacted with REM percentage: higher REMS percentage associated with less overnight SCR habituation *via* non-continuous REMS. This pattern resembles a previous finding, where the habituation effect of REMS was lost in case of abundant interruptions (Wassing et al., 2019a). We did not find SWS to be associated with any habituation outcome. Experimentally disrupting these sleep stages yielded comparable results (Glosemeyer et al., 2020), supporting REMS as the primary sleep stage for affective processing.

What is adaptive emotional processing? Preserving affective load ensures readiness to respond rapidly under threat. However, allowing irrelevant non-threats to control nighttime recovery is equally disruptive. From this perspective, we assessed participants for their trait-like tendency to experience shame. This trait was associated robustly with both physiological and subjective affective responses in our data (unlike other questionnaire scores), and it moderated the association between REM fragmentation and SCR habituation. Attenuated post-sleep SCR was observed in highly shame-prone individuals as a function of less REM fragmentation, the pattern being opposite in those with a low tendency to experience shame. This finding may reflect an adaptive nature of (consolidated) REMS, as it appears to preferably scale down the affect in those easily overridden by a shameful event. A completely effacing affective response is not adaptive either, perhaps reflected in preserved SCR in low-shame-prone individuals. Individual propensity should be considered in research deploying stressors that may be unequally experienced.

Notably, subjective response was not associated with either SCR or sleep parameters. Particularly, social stress may evoke uncorrelated objective and subjective responses (Mauss et al., 2004) relative to non-social stress (Reinhardt et al., 2012). Self-report is prone to cognitive appraisal and may dispel positive arousal more readily. We also observed subjective responses nearing a floor effect, several participants rating low embarrassment already in the pre-sleep assessment, compromising statistical resolution.

Piloting the TMR approach did not indicate differences in affective habituation. This diverges from a study where odor-bound TMR attenuated amygdala response toward a similar stressor (Wassing et al., 2019a). While an obvious difference from our study was their use of odors instead of sounds, TMR-enhanced affective habituation has also been shown using sounds (He et al., 2015; Hutchison et al., 2021). The properties of our TMR procedure probably underlie the negative results. First, the inter-cue interval was 1.5 s, confounding with the refractory period of a possible post-cue spindle during SWS and impairing processing (Antony et al., 2018). Second, the amount of cueing requires consideration. While repeated replays may reduce the excitability of concerned cortical networks (Lewis and Bendor, 2019), experimental evidence suggests that increasing the amount of cueing inflicts a stronger effect (He et al., 2015). Our less-intensive TMR was probably insufficient to cause observable effects.

### Strengths and Limitations

In this study, we investigated the role of REMS as a modulator of emotional processing. We contributed to the emerging understanding that specifically fragmented REMS may underlie maintained stress response overnight. Moreover, considering the individual predisposition to experience self-conscious stress, we also show that this trait may modulate how REMS quality relates to the post-sleep response.

There are significant limitations to be considered. Mainly, the sample was small, decreasing statistical power and increasing the propensity for confounding factors. Observing a few participants resilient to the stress induction further highlights this limitation. While aiming at a statistically significant TMR effect likely requires a larger sample, the obtained effect sizes indicate that it would not have impacted the TMR outcome in our study. Along these lines, the TMR setting was possibly insufficiently intense to induce consequential differences in a neural replay, necessitating further undertakings to elucidate TMR's applicability in social stress. Regarding other findings, the significant results were correlational, precluding causal deductions. Measuring only one night disallows the examination of within-subject changes in relevant REMS parameters. Finally, we did not contrast the response to their own-singing-response with a response caused by hearing someone else's singing, partially disputing the response being related to the self-conscious affect. This is mitigated by the strong associations between the observed response and trait-like shame.

### Conclusions

The potential of sleep in affective processing has gathered both interest and evidence in recent years. While we did not find TMR protocol to influence overnight habituation for self-conscious affect, we observed REMS and its fragmentation to be associated with post-sleep response intensity. Frequent interruptions in REMS can have adverse effects on emotional downscaling. Moreover, the need for this habituation, reflected by the trait of shame-proneness, may moderate how consolidated REMS scales the response.

## Data Availability Statement

The raw data supporting the conclusions of this article will be made available by the authors on request, without undue reservation.

## Ethics Statement

The studies involving human participants were reviewed and approved by Ethics Committee of Helsinki University Hospital. The patients/participants provided their written informed consent to participate in this study.

## Author Contributions

RH: conceptualization, methodology, writing—original draft preparation, formal analysis, investigation, and visualization. LK: conceptualization and review and editing. TM: technical preparation of skin conductance measurement and analysis. JK: set-up of karaoke recording and playback system. A-KP: conceptualization, review and editing, supervision, and project administration. All authors contributed to the article and approved the submitted version.

## Funding

This study was supported by the Academy of Finland (Grant No. 1322035) and Signe och Ane Gyllenberg Foundation.

## Conflict of Interest

The authors declare that the research was conducted in the absence of any commercial or financial relationships that could be construed as a potential conflict of interest.

## Publisher's Note

All claims expressed in this article are solely those of the authors and do not necessarily represent those of their affiliated organizations, or those of the publisher, the editors and the reviewers. Any product that may be evaluated in this article, or claim that may be made by its manufacturer, is not guaranteed or endorsed by the publisher.
